# Co-infection by human immunodeficiency virus type 1 (HIV-1) and human T cell leukemia virus type 1 (HTLV-1): does immune activation lead to a faster progression to AIDS?

**DOI:** 10.1186/1471-2334-9-211

**Published:** 2009-12-22

**Authors:** Eduardo Samo Gudo, Nilesh B Bhatt, Dulce Ramalho Bila, Celina Monteiro Abreu, Amílcar Tanuri, Wilson Savino, Suse Dayse Silva-Barbosa, Ilesh V Jani

**Affiliations:** 1 Department of Immunology, National Institute of Health, Maputo, Mozambique; 2 Laboratory on Thymus Research, Oswaldo Cruz Institute, Oswaldo Cruz Foundation, Rio de Janeiro, Brazil; 3 Departament of Genetics, Federal University of Rio de Janeiro, Rio de Janeiro, Brazil; 4 Center for Bone Marrow Transplantation, National Cancer Institute, Rio de Janeiro, Brazil

## Abstract

**Background:**

Recent data have shown that HTLV-1 is prevalent among HIV positive patients in Mozambique, although the impact of HTLV-1 infection on HIV disease progression remains controversial. Our aim was to determine the phenotypic profile of T lymphocytes subsets among Mozambican patients co-infected by HIV and HTLV-1.

**Methods:**

We enrolled 29 patients co-infected by HTLV-1 and HIV (co-infected), 59 patients mono-infected by HIV (HIV) and 16 healthy controls (HC), respectively.

For phenotypic analysis, cells were stained with the following fluorochrome-labeled anti-human monoclonal antibodies CD4-APC, CD8-PerCP, CD25-PE, CD62L-FITC, CD45RA-FITC. CD45RO-PE, CD38-PE; being analysed by four-colour flow cytometry.

**Results:**

We initially found that CD4^+ ^T cell counts were significantly higher in co-infected, as compared to HIV groups. Moreover, CD4^+ ^T Lymphocytes from co-infected patients presented significantly higher levels of CD45RO and CD25, but lower levels of CD45RA and CD62L, strongly indicating that CD4^+ ^T cells are more activated under HTLV-1 plus HIV co-infection.

**Conclusion:**

Our data indicate that HTLV-1/HIV co-infected patients progress with higher CD4^+ ^T cell counts and higher levels of activation markers. In this context, it is conceivable that in co-infected individuals, these higher levels of activation may account for a faster progression to AIDS.

## Background

Infection by the Human Immunodeficiency Virus (HIV) has been considered a serious infectious disease particularly in Southern Africa, which harbors more than 2/3 of all worldwide cases of HIV [1].

The emergence of several co-pathogens has aggravated this scenario in resource-limited settings [2,3]. Human T-lymphotropic virus type 1 (HTLV-1) has been implicated as a frequent co-pathogen in areas or groups where both viruses are prevalent [4,5]. In the southern Africa region where HIV is highly prevalent [6], the prevalence of co-infection by HTLV-1 and HIV varies among countries and in several places have been reported be higher than 10% [7-9]. A recent study conducted in Mozambique reported a co-infection prevalence rate of 4.5% among HAART naïve HIV positive individuals. Thus, it is conceivable that the impact of chronic infection by HTLV-1 on HIV disease progression is a relevant issue in AIDS research. Nevertheless, such an issue remains controversial, and published data are conflicting[10-12]. If in one hand it was initially postulated that co-infected patients progress faster to AIDS[13], further studies reported contradictory results[10,14].

The influence of HTLV-1 on HIV disease progression has been tightly linked, not only to several molecular events [15,16] but to its potential to induce high levels immune activation [17]. Although the mechanisms by which chronic activation induced by HTLV-1 could potentially affect the progression to AIDS are not completely understood, we have learned from the natural history of HIV infection that chronic activation of the immune system takes part and triggers a number of cellular and molecular pathways related to CD4^+ ^T cell loss and immune deregulation [18-22]. In this context, HTLV-1 per se induces a strong immune activation [23,24] that has been associated to immunosuppression, unresponsiveness and immune deregulation [25,26]. How the immune system behaves in the presence of both HIV and HTLV-1 remains to be clarified.

The concern regarding the clinical outcome as a result of co-infection by HTLV-1 and HIV has gained a special relevance in recent years, in face of the growing body of evidence showing that: a) co-infection is prevalent in several geographical regions in Southern Africa [27,28]; b) we and others showed that co-infected patients present stable CD4^+ ^T lymphocytosis irrespective of their progression to AIDS, which by certain extent could mask the immunosuppression with consequent inappropriate therapeutic decisions in terms of the initiation of Highly Active Anti Retroviral Therapy (HAART) and prophylaxis for opportunistic infections [29,30].

The situation has being aggravated by the fact that neither cure nor effective treatment is yet available for HTLV infection [31] in such a way that clinicians are unable to control the effects exerted by the virus.

Previous studies have enrolled patients with different HAART experiences and some authors believe that this may partially explain the divergence of results obtained [11]. Other studies were based on *in vitro *manipulation [15] or used simian models [32]. In addition, no study has been so far conducted in Africa, the region carrying the greatest burden of HIV disease and where the epidemiology of HIV and other diseases is quite different [1].

Altogether, these data raise the need to define to what extent HTLV-1 impacts the clinical progression to AIDS in an African setting. We evaluated herein HTLV-1 and HIV co-infected HAART naïve adult patients, in terms of T cell phenotype, further correlating the expression of activation markers and HIV-1 viral load. We showed that co-infected patients progress with higher levels of CD4^+ ^T cells expressing activation markers and a massive loss of naïve cells, thus suggesting that co-infected patients progress faster to AIDS.

## Methods

### Study design and subjects

A case control study was conducted with participants consisting of three sets, namely, individuals co-infected by HIV and HTLV-1 (Co-infected), HIV mono-infected patients (HIV) and healthy controls (HC). Co-infected and HIV groups were recruited from an ongoing cohort of HIV infected patients followed in the Alto Maé Health Centre, in the city of Maputo, Mozambique. In the period between March and June 2006, 724 HIV 1/2 infected patients were invited to participate and 704 (97.4%) accepted to be part of this study. They were all screened for HTLV-1 infection and 32 patients (4.5%, 32/704) were founded to be co-infected by HTLV-1 and HIV. Three patients with a positive HTLV-1 antibody test did not return to collect their result and were excluded of the study. Co-infected were matched at a ratio 1:2 with HIV mono-infected by age, sex and HIV clinical stage system as defined by WHO [33]. Co-infected and HIV mono-infected were matched without prior knowledge of CD4+T cell counts results.

Healthy controls were not matched by age and sex as we did with co-infected and HIV-mono-infected, since they were recruited on a consecutive basis from the routine blood donors at the blood bank of Maputo Central Hospital. In addition, most of blood donors are males and younger as demonstrated by two previous studies conducted at the same Blood Bank ([34,35]) Informed consent to participate in the study was requested to all participants, and the study was approved by the National Bioethics Committee in Mozambique and by the Sydney University Ethics Committee, Australia.

Physical and neurological examination was performed by two medical doctors blinded for the HTLV-1 status. Socio-demographic data, sexual/reproductive history and clinical data were also recorded from each participant.

The study population consisted of 59 HIV, 29 co-infected and 16 healthy controls individuals. All HIV and co-infected individuals were naïve to HAART. The median age was 40 years (IQR, 34 - 48 years) for co-infected individuals, 41 years (IQR, 32 - 47) for HIV patients and 32 (IQR, 29 - 38) for HC, respectively. There was a predominance of the female gender among HIV and co-infected individuals (86,3% and 86.4% respectively) and predominance of the male gender among HC (80%; see table 1). Almost half of co-infected and HIV patients were classified as stage 2 for HIV clinical disease (58.4% and 56.0% for co-infected and HIV patients respectively) based on the WHO criteria [33]. None of the co-infected or HIV patients was at stage 4 of HIV clinical disease (table 1). HAM/TSP or ATL cases were not detected in subjects of any groups.

**Table 1:** General characteristics of HIV/HTLV-1 co-infected, HIV mono infected and healthy Mozambican subjects

General Features	HIV/HTLV-1 (n = 29)	HIV (n = 59)	HC (n = 16)	*p value*
**Age, years**				
Median	40.0	41.0	32.0	
IQR	34.0 - 48.0	32.0 - 47.0	29.0 - 38.0	0.123*
**Gender**				
Male	4 (13.8%)	8 (13.6%)	12 (80%)	
Female	25 (86.3%)	51 (86.4%)	3 (20%)	0.000**
HIV clinical stage***				
I	7 (24.1%)	14 (23.7%)		
II	17 (58.4%)	33 (56.0%)		
III	5 (17.2%)	12 (20.3%)		0.587**

* One Way Anova; ** Chi square; ***as defined by WHO[30];

HIV/HTLV-1, HIV/HTLV-1 co-infected; HIV, HIV mono-infected; HC, healthy controls

Co-infected and mono-infected were comparable regarding clinical presentation of opportunistic diseases (table 2).

**Table 2:** Clinical presentation of study groups

Clinical presentation	HIV/HTLV-1, n(%) (n = 29)	HIV, n(%) (n = 59)	p value*
**Assymptomatic**	7 (24.1)	14 (23.7)	
**Papular pruritic eruptions**	3 (10.3)	14 (23.7)	
**Dermatitis**	2 (6.9)	2 (3.4)	
**Seborrhoeic dermatitis**	2 (6.9)	0 (0.0)	
**Folliculitis**	0 (0.0)	1 (1.7)	
**Herpes zoster < 5 years**	3 (10.3)	4 (6.8)	0.570
**TInea capitis**	5 (17.2)	8 (13.6)	
**Candidiasis**	3 (10.3)	4 (6.8)	
**Tuberculosis < 1 year**	1 (3.5)	4 (6.8)	
**Weight loss > 10%**	3 (10.3)	7 (11.9)	
**Chronic diarrhoea**	0 (0.0)	1 (1.7)	

* Fisher chi square test

### Blood samples

Ten milliliters of venous whole blood were requested from each volunteer. The blood was collected aseptically into a 5 ml vacuum tube with K_3_EDTA and a 5 ml vacuum tube for serum separation (Becton-Dickinson Vacutainer Systems, USA). Blood specimens were delivered at the laboratory within four hours of collection.

### HIV Serology

All patients enrolled in this study were screened for anti-HIV 1/2 antibodies at the Voluntary Counseling and Testing (VCT) services of the Mozambican Health Center. Patients were tested for HIV according to the Mozambican National protocol consisting of a sequential algorithm of two immunochromatographic rapid tests. All individuals were first screened using the Determine HIV-1/2 test (Abbott Laboratories, Japan). All specimens reactive on the screening assay were further tested using the Uni-Gold HIV test (Trinity Biotech, Ireland). Individuals reactive on both assays were considered positive for HIV-1/2 infection.

### HTLV Serology

All samples were screened for anti-HTLV-1+2 antibodies using the qualitative EIA Murex HTLV-1 + 2 (Murex Biotech Limited, UK). Specimens reactive on the EIA were confirmed by a Western blot assay (HTLV BLOT 2.4, Genelabs^® ^Diagnostics, Switzerland). All patients with reactivity to antigens encoded by the GAG gene (p19 with or without p24) and to two antigens encoded by the ENV gene (GD21 and rgp46-I) were considered to be infected by HTLV-1 according to the instructions provided by the manufacturer. All HTLV positive samples in our study population were typed as HTLV-1 by Western blot.

### T cell immunophenotyping

Phenotypic analysis of circulating T lymphocytes was performed through four colour flow cytometry on fresh EDTA-anticoagulated whole blood using a FACSCalibur™ flow cytometer (Becton-Dickinson Biosciences, USA). T cells counts were obtained based on a lyse-no wash protocol using CellQuest Software for acquisition (BD Biosciences). Cells were stained with the following fluorescent-labeled anti-human monoclonal antibodies CD4-APC, CD8-PerCP, CD25-PE, CD62L-FITC, CD45RA-FITC. CD45RO-PE, CD38-PE (all from BD Biosciences). Analyses were performed using the Summit software v 4.3.2, 2006 (Dako Cytomation, Inc., Fort Collins, USA).

### HIV-1 subtyping

Samples from co-infected and mono-infected individuals were sequenced for HIV-1 subtyping. A 297-bp fragment encompassing full sequence of the protease gene was amplified using nested PCR. The outer primers PRT15F 5' TGAAAGATTGTACTGAGAGACAGG 3'/K2R 5' GTCAATGACATACAGAAGTTAGTGGGAAAA 3' were used in first-round PCR, and DP10F 5' CAACTCCCTATCAGAAGCAGGAGAAG 3'/RVP3R 5'-CCATACAATACTCCAGTATTTGCC-3' were used as inner primers during the second-round of nested PCR. The conditions for both rounds of PCR were described previously [36]. Amplified DNA was quantified, purified and sequenced under PCR conditions described elsewhere [37], using the primers from the second-round PCR. The genetic subtypes were determined using the amino acid sequences of the protease genes, deduced from the nucleic acid sequences, and were compared to a subtype B consensus sequence from the Stanford HIV Protease Sequence database http://hivdb.stanford.edu/hiv/.

The analysis of the Mozambique sequence with HIV Drug Resistance database showed that all samples sequenced (20 mono-infected and 24 co-infected) were subtype C in protease gene with profile of similarity of 98% compared with sequence subtype C from the Stanford HIV Protease Sequence database.

### Stool samples

Stools were collected into a sterile, wide mouth, leak-proof container with a tight fitting lid containing a preservative solution. Stools were kept at room temperature until delivery at the study setting within six hours of collection. Small amounts of stool specimens were placed on microscope slides and mixed with 0.9% NaCl solution to prepare wet mount smears. Slides were then examined under a light microscope to screen for cysts of *Giardia lamblia*, *Entamoeba hystolitica*, *Entamoeba coli *and *Balantidium coli*, ova of *Ascaris lumbricoides*, *Trichuris trichiura *and *Ancylostoma duodenale *and larvae of *Strongyloides stercolaris*.

### Statistical analysis

Data was analyzed using the statistics package STATA 9.0 (College Station, Texas: StataCorp, USA, 2005).

Taking into consideration that the major goal of our study was to compare activation markers between co-infected *versus *HIV mono-infected patients, two sample comparison means with a ratio 1:2 (case:control) was used to determine the required sample size in these groups. Due to lack of information regarding comparison of activation markers between these groups, we calculated a sample size enough to detect a least a difference of 10% in the mean of these cells frequencies with a standard deviation of 14 at a significance level of 5%.

The Mann-Whitney test and the One Way Anova trend test were used to compare the differences among numerical variables in the three groups. Associations between categorical variables were determined using the Pearson Chi-square test, the Fisher exact test or the Chi-square trend test, as appropriated.

## Results

### HIV/HTLV-1 co-infected individuals present higher and stable CD4^+ ^T cell counts

**Figure 1:**
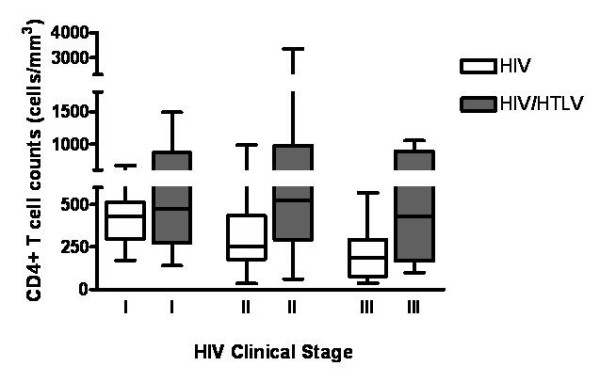
**Absolute CD4+ T cell counts in different HIV clinical stages, among different HTLV status**. There is a consistent and stable high absolute CD4 T cell count in co-infected individuals, contrasting with the progressive decrease of this cell subset in the monoinfected group, related to the progression of HIV disease. p value for Anova trend test was 0.000 and 0.945 for HIV/HTLV-1 and HIV groups respectively.

We first showed that HIV/HTLV-1 co-infected individuals exhibited a higher absolute and relative CD4^+ ^T cell counts (median: 525 cells/mm^3^, versus 274 cells/mm^3^, p = 0.000 and 24.9% versus 15.9%, p = 0.000, see table 3) and a higher CD4^+^/CD8^+ ^T cell ratio (0.5 versus 0.30, p = 0.004), when compared with the HIV mono-infected group. Moreover, CD4^+ ^T lymphocytosis in co-infected individuals was stable irrespective of their HIV clinical stage, thus contrasting with the gradual loss of CD4^+ ^T cells from HIV clinical stage I through stage IV seen in the mono-infected group (figure 1). Both groups were similar regarding CD8^+ ^T cell absolute counts but co-infected individuals presented lower CD8^+ ^T cell relative counts (p = 0.505 and p = 0.009, respectively; see table 3). These patterns were the same in both males and females (data not shown).

**Table 3:** Distribution of T cell subsets in peripheral blood from HIV/HTLV-1 co-infected, HIV mono-infected and healthy Mozambican subjects

T cell subsets and viral load	HIV/HTLV-1 (n = 29)	HIV (n = 59)	HC (n = 16)
**CD4^+ ^T cell counts (cells/mm^3^)**			
Median	525^a^	274	472
IQR	310 - 827	183 - 436	412 - 775
**CD4^+ ^T cell counts (%)**			
Median	24.9 ^a, b^	15.9	45.0
IQR	19.0 - 32.7	9.4 - 21.0	37.0 - 48.0
**CD8^+ ^T cell counts, cells/mm^3^**			
Median	1002^b^	937	302
IQR	649 - 1090	606 - 1358	190 - 391
**CD8^+ ^T cell counts, %**			
Median	46.8^a, b^	54.2	23.0
IQR	36.2 - 53.0	42.7 - 61.3	21.0 - 29.0
**CD4^+ ^T/CD8^+ ^T ratio**			
Median	0.5^a, b^	0.3	1.8
IQR	0.3 - 0.8	0.2 - 0.4	1.2 - 2.5
**CD8^+ ^CD38^+ ^T cells (MFI)**			
Median	341.5	279.9	209.7
IQR	285.1 - 367.1	225.4 - 373.3	209.7 - 225.4
**HIV-1 RNA viral load (copies/mL)**			
Median	56,385	37,573	
IQR	14,749 - 277,570	11,322-176,837	

One ANOVA corrected by Bonferoni was used to compare groups.

HIV/HTLV-1, HIV/HTLV-1 co-infected; HIV, HIV mono-infected; HC, healthy controls; MFI, Median Fluorescence Intensity

^a ^p < 0.05 comparison co-infected versus HIV mono-infected

^b ^p < 0.05 comparison co-infected versus healthy controls

### Higher proportions of CD4^+ ^T cells bearing an activated phenotype in HIV/HTLV-1 co-infected individuals

**Figure 2:**
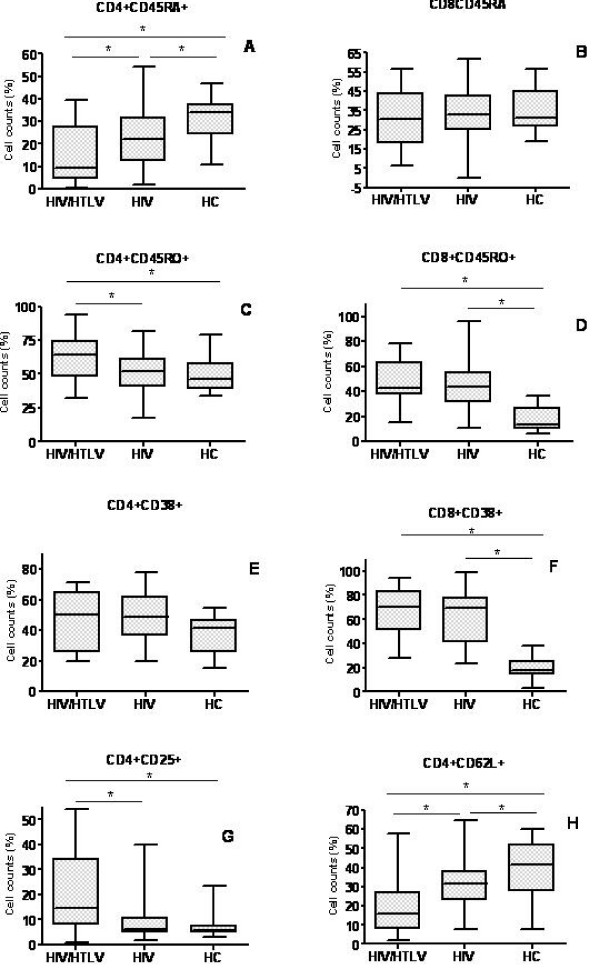
**Distribution of T cell subsets in HIV/HTLV-1 co-infected, HIV mono-infected and healthy Mozambican subjects**. Co-infected patients presented higher CD4+CD45RO+ (*memory cells*, **C**), CD8+CD38+ (*activated cells*, **E**) and lower CD4+CD45RA+ (*naïve cells*, **A**) and CD4+CD62L+ (*naïve cells*, **H**). *p < 0.05, as ascertained by One Way Anova. **HIV/HTLV **= coinfected, **HIV **= mono-infected by HIV and **HC **= healthy controls.

**Figure 3:**
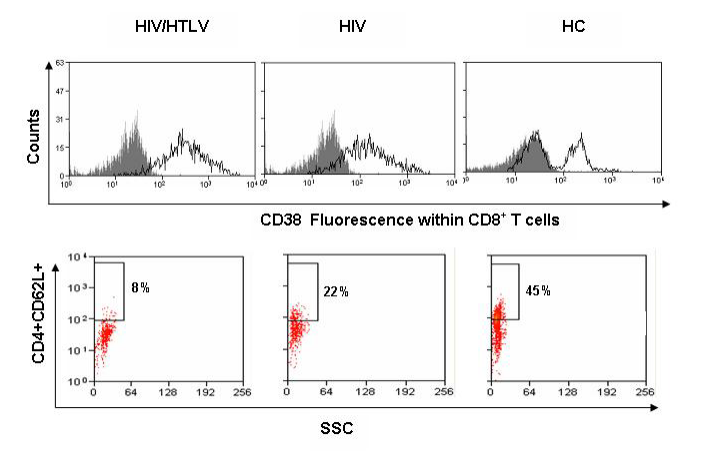
**Activated and naïve T cell profiles HIV/HTLV-1 co-infected, HIV mono-infected and healthy Mozambican subjects**. Dot plots show the typical phenotype of activated (upper panels) and naïve T lymphocytes (lower panel). Upper panels reveal that co-infected patients presented with higher density of CD38 molecules on T CD8+ T cells. Co-infected patients presented lower frequency of CD4^+^CD62L^+ ^cells when compared with HIV positive patients and health controls (lower panel).

The data described above, on the differences in CD4^+ ^T cell counts between co-infected *versus *HIV patients, pointed to a differential activation state of T cells in these patients. In fact, when compared to HIV or to HC participants, co-infected individuals presented significantly higher membrane levels of CD25 and CD45RO on CD4^+ ^T cells (0.007 and 0.040 respectively, figure 2C and 2G). Also the density of CD38 molecules on the surface of CD8^+ ^T cells was higher in co-infected when compared to HIV and HC participants, although not statistically significant, (figure 3 and table 3). The relative numbers of CD8^+^CD38^+ ^and CD8^+^CD45RO^+ ^cells in co-infected although statistical significantly higher than HC (p = 0.000 for both CD8^+^CD38^+ ^and CD8^+^CD45RO^+ ^respectively, figures 2D and 2F) was only slightly higher than HIV individuals (median: 51.3%, versus 41.2%, p = 0.652 for CD8^+^CD38^+ ^and 37.4% versus 31.0%, p = 0.512 for CD8^+^CD45RO^+ ^respectively, figures 2D and 2F).

All three groups were similar regarding the expression of CD38 on CD4^+ ^T cells (figure 2E).

Naïve cells were phenotyped for the expression of CD45RA and CD62L on CD4^+ ^and CD8+ T cells respectively. Co-infected and HIV individuals exhibited lower levels of CD45RA (p = 0.023 and 0.037 respectively) and CD62L (p = 0.026 and 0.041 respectively) on CD4^+ ^T lymphocytes, when compared to HC, Such a loss of naïve cells was more pronounced in the co-infected group (figures 2A, H and 3).

Of interest, stool evaluation revealed no significant differences in helminthic or protozoan loads among the three groups of patients (data not shown), indicating that the relative role of parasites in inducing lymphocyte activation is likely similar in the various groups.

### Activation markers on T cells correlate with HIV clinical stage

**Figure 4:**
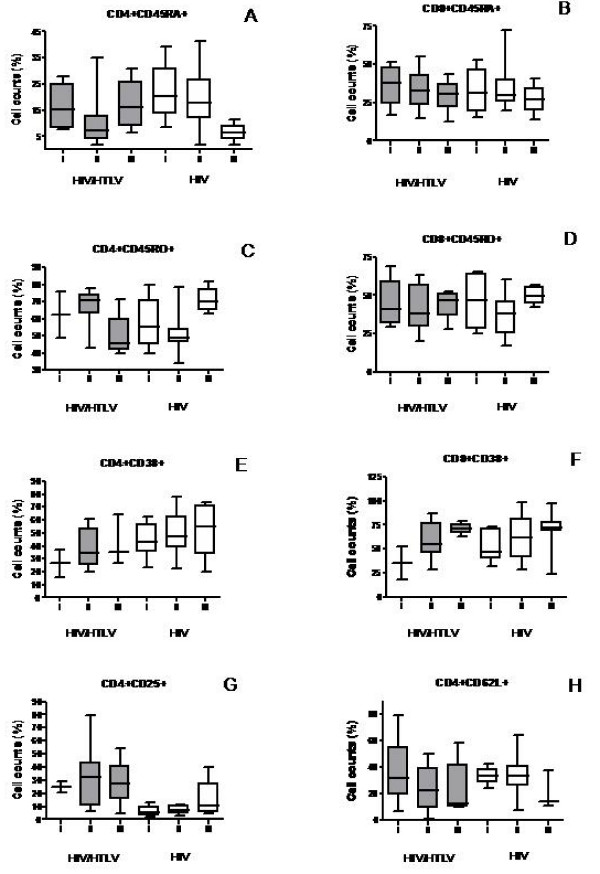
**Changes in the subsets of T cells in the distinct HIV clinical stages**. CD8+CD45RO+ (D) remained unchanged in the various HIV clinical stages in both groups. CD38+ on CD4+ and CD8+ T cells (E and F), CD4+CD25+ and CD4+CD45RO+ subsets were increased from clinical stage I through III in both groups. CD4+CD45RA+ (A), CD8+CD45RA+ (B) and CD4+CD62L+ (H) decreased from stage I through III. Co-infected patients in the clinical stage III for CD4+CD25+ and CD4+CD45RO+ and CD4+CD45RA+ did not follow the pattern observed in HIV mono-infected individuals.** I **= HIV Clinical Stage I, **II **= HIV Clinical Stage I, ** III **= HIV Clinical Stage III.

The small sample size of our study was a limitation to assess the changes in the activation by HIV clinical stage. Therefore, although a statistical analysis was not possible to perform, we stratified the analysis of these subsets by HIV clinical stage in an intent to define a pattern (figure 4). Our data found that the relative numbers of CD8+CD45RO+ (figure 4D) T cells remained unchanged from HIV clinical stages I to III. By contrast, there was an increase in the expression of CD38 in both CD4+ and CD8+ T cell subsets (figures. 4E-F) and a decrease of CD4+CD62L+ and CD8+CD45RA+ (figure 4B) subsets from clinical stage I through III in both groups. The frequency of CD4+CD45RO+ (figure 4A) CD4+CD25+ (figure 4G) subsets was higher from stage I through III among HIV mono infected patients. Rather unexpectedly, among co-infected patients the frequencies of these subsets were lower in the clinical stage III when compared to clinical stage II. Similarly in respect to the frequency of the CD4+CD45RA+ subset (figure 4A) there was an decrease from stage I through stage III among HIV monoinfected patients. Surprisingly, among co-infected patients, the frequency of cells in the clinical stage III was higher than in the clinical stage II. It is possible that the small sample size in each clinical stage may explain these unexpected findings

### HIV-1 viral load positively correlates with the enhancement of T cell activation markers in both co-infected and HIV groups

**Figure 5:**
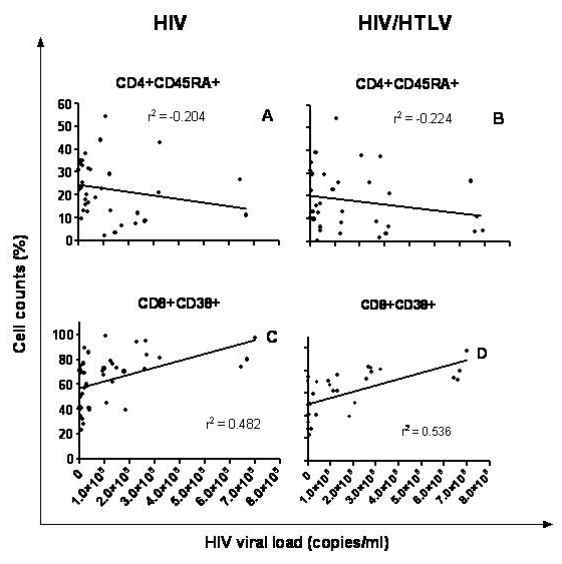
**Correlation between naïve CD4 (CD4^+^CD45RA^+^) or activated CD8 (CD8^+^CD38^+^) T lymphocytes with HIV-1 viral Load**. Panels A and B depict a negative correlation between CD4+CD45RA+ (naive cells) and HIV-1 viral load in both co-infected and HIV-1 subjects. By contrast, lower panels show a positive correlation between CD8+ CD38 (activated cells, **C **and **D) **and HIV-1 viral load in both co-infected and HIV-1 subjects.

Although weak, there was an inverse correlation between the proportions of CD4^+^CD45RA^+ ^naïve cells and HIV-1 viral load. Co-infected group appeared to present a better correlation when compared to HIV group (r = -0.224 *versus *-0.204 respectively, figures 5A-B), but the difference was not statistically significant.

In contrast, there was a positive correlation between the proportions of CD8^+^CD38^+ ^cells and HIV-1 viral load. Again co-infected group presented a slightly higher correlation, but such a difference was not statistically significant (r = 0.536 *versus *0.482 respectively, figures 5C-D).

## Discussion

HIV and HTLV-1 have emerged as common co-pathogens especially in areas or groups where both viruses are circulating [5,9,38,39]. Nevertheless, the impact of HTLV-1 on HIV disease progression is still a matter of debate with controversial results [10-14,30,40]. Here, for the first time we conducted a case control study in an African setting, aiming to determine the impact of HTLV-1 infection on HIV disease. In fact, to our knowledge, the present study is pioneer in the region, since it was conducted in HAART naïve patients, on a well controlled cross-sectional basis.

Previous studies were conducted mainly in South and North Americas where the epidemiology of HIV infection and other diseases is quite different from that seen in sub-Saharan African countries [29,30].

We found no evidence of HTLV-2 in our study population. This is in keeping with two recent studies conducted among blood donors in Maputo city[34,35] and suggest that only HTLV-1 (but not HTLV-2) circulates in Mozambique.

As expected, co-infected individuals presented a stable CD4^+ ^T lymphocytosis irrespective of their progression to AIDS, contrasting with the depletion of CD4^+ ^T cells counts observed among HIV patients over time. To date, it is well established that cell immortalization and transformation induced by Tax and Rex proteins encoded by HTLV-1 genes constitute major events related to uncontrolled CD4^+ ^T cell growth and proliferation [41,42].

The intriguing progression to AIDS in the presence of normal or high levels of CD4+T cells counts suggest these to be functionally altered. Consensus exists that both HIV [43,44] and HTLV [24,25,45] separately induce functional modifications on T cells populations, characterized among others by a decrease of naïve populations and higher levels of cell activation when compared with uninfected individuals.

Here we found that co-infected individuals presented markedly lower expression of CD45RA^+ ^(a phenotypic marker of naïve T lymphocytes) on CD4^+ ^T cells. Naïve cells are considered the first cells to be depleted in the presence of immune activation [46,47] and represent one of the hallmarks of HIV infection [48]. The magnitude and impact exerted by naïve T cells erosion on HIV disease progression remain to be defined. Although not fully understood, there is a consensus that for both HIV and HTLV-1, the loss of naïve cells has been linked, among others, to, (i) a homeostatic mechanism to replenish the cells being killed (ii) a massive recruitment of naïve cells, partially imposed by the mechanisms driving the activation and (iii) the impairment of T cell production [19,20,46-49].

In our study, the erosion of the naïve compartment was further confirmed by evaluating the expression of CD62L, another marker for naïve T lymphocytes, usually lost upon activation. As expected, there was a dramatic loss of CD4^+^CD62L^+ ^lymphocytes in the co-infected group, when compared to HIV mono-infected and HC groups. Importantly, these differences were further confirmed when we compared the groups in terms of naive cell absolute counts (data not shown) arguing against an indirect effect of higher percent counts of memory cells. Whether there is an impairment of T cell production, if they are dying faster or if more cells being recruited from the naïve T cell pool into activated/memory cell compartment remain to be determined.

Not surprisingly, this loss of naïve cells in co-infected individuals was accompanied by higher frequencies of memory and activated cells as measured by CD45RO^+ ^(memory), CD38^+ ^and CD25^+ ^(activated) cell markers. In fact, co-infected individuals presented with higher proportions of CD45RO^+ ^on CD4^+ ^T cells when compared to the HIV and HC groups. These findings are in agreement with previously data [45,46,50,51], Similarly, the relative number of CD4^+^CD25^+ ^cells seen in co-infected patients was higher than what was found in HIV and HC individuals. It is conceivable that the increase of CD4^+^CD25^+ ^cells is a consequence of the virus-driven induction of IL-2/IL-2 receptor expression by tax, as previously reported [12,23,52]. Interestingly, the frequency of CD38^+^cells within the CD8^+ ^T cell compartment but not in CD4^+ ^T cells was increased in co-infected and HIV when compared to HC. This is in keeping with the results showed in a case-control study conducted among HAM/TSP patients [24]. Although we did not find differences in the frequency of CD38^+ ^cells, either in CD8^+ ^or CD4^+ ^T cells, we found that co-infected patients presented higher expression of CD38 in CD8^+ ^T cells (as ascertained by Median Fluorescence Intensity measurements) when compared to HIV patients. Nevertheless differences were not statically significant.

Noteworthy, increased expression of CD38 on the surface of CD8^+ ^T cells have long been considered an even better prognostic predictor of progression to AIDS and response to HAART than HIV viral load itself [53,54]. This is relevant due to the fact that such parameter is being proposed to be included in clinical settings to monitor HIV disease progression [55].

It is now widely accepted that the presence of chronic activation is a major factor influencing the pathogenesis of HIV in Africa [56]. HTLV-1 is a strong activator of immune system. Immune activation and exaggerated immune response has been demonstrated to be the main pathogenetic mechanism involved in the HTLV-1 associated inflammatory syndromes[24,57-59]. The immunodominant Tax protein encoded by HTLV transactivates and modulates a large number of genes playing a key role in triggering several pathways leading to cell activation[60-62]. Available data demonstrate that a large proportion of asymptomatic carries progress with high levels of immune activation[63].

On the basis of the patients' age and their HAART naïve status, we believe that HTLV-1 infection preceded HIV infection. Considering that individuals chronically infected by HTLV progress with immune activation, it is conceivable that these patients acquire HIV infection in a pre-activated immune milieu, and the presence of immune hyper activation not only turns them more susceptible to acquire HIV, but also to progress faster to a poor prognosis.

Cases and controls were matched by age and clinical stage (WHO) so that to be comparable in terms of clinical presentation (see table 2), Clinical staging system is performed on the basis of patient's clinical presentation. This information is important when interpreting the differences in the activation markers between these groups. Another aspect deserving discussion is the helminthic infection as a factor involved in immune activation, particularly in Southern Africa [3,64]. Accordingly, a differential presence of parasitic infection in our patients could bias our results. However, this does not seem to be the case since in all groups evaluated, the degree of protozoan and helminthic infections were similar. Of note, all samples sequenced in both groups were founded to be HIV subtype C, ruling out any linkage between HIV subtype in mono and co-infected groups, and immunological/clinical behavior.

## Conclusion

In conclusion, although HIV/HTLV-1 co-infected individuals quantitatively maintain a normal or high CD4^+ ^T cells counts, these cells are likely functionally altered presenting with a dramatic decrease of naïve cells and higher activation patterns. Yet, if these changes account for a faster progression to AIDS remains to be determined.

## Competing interests

The authors declare that they have no competing interests.

## Authors' contributions

ESG participated in the study design, data collection, data analysis, and writing the manuscript. NBB participated in the study design, data collection, data analysis and writing the manuscript. DRB participated in the study design, sample processing and data analysis. WiS participated in the study design, data analysis and writing the manuscript. SDSB participated in the study design, data analysis and writing the manuscript. CMA and AT participated in HIV subtyping and writing the manuscript. IVJ participated in the study design, data collection, data analysis and writing the manuscript. None of the authors have any financial or personal interest in the World Bank Quick Impact Fund. All authors have read and approved the final manuscript

## Pre-publication history

The pre-publication history for this paper can be accessed here:

http://www.biomedcentral.com/1471-2334/9/211/prepub
